# Calcitriol Alleviates MPP^+^- and MPTP-Induced Parthanatos Through the VDR/PARP1 Pathway in the Model of Parkinson’s Disease

**DOI:** 10.3389/fnagi.2021.657095

**Published:** 2021-07-30

**Authors:** Junjie Hu, Jiawei Wu, Fang Wan, Liang Kou, Sijia Yin, Yadi Sun, Yunna Li, Qiulu Zhou, Tao Wang

**Affiliations:** Department of Neurology, Union Hospital, Tongji Medical College, Huazhong University of Science and Technology, Wuhan, China

**Keywords:** Parkinson’s disease, calcitriol, parthanatos, VDR, PARP1

## Abstract

The pathogenesis of Parkinson’s disease (PD) is currently unclear. Recent studies have suggested a correlation between vitamin D and PD. Vitamin D and its analogs have protective effects in animal models of PD, but these studies have not clarified the mechanism. Parthanatos is a distinct type of cell death caused by excessive activation of poly (ADP-ribose) polymerase-1 (PARP1), and the activation of PARP1 in PD models suggests that parthanatos may exist in PD pathophysiology. 1,25-Dihydroxyvitamin D3 (calcitriol) is a potential inhibitor of PARP1 in macrophages. This study aimed to investigate whether calcitriol treatment improves PD models and its effects on the parthanatos pathway. A 1-methyl-4-phenylpyridinium (MPP^+^)-induced cell model and 1-methyl-4-phenyl-1,2,3,6-tetrahydropyridine (MPTP) subacute animal model were selected as the *in vitro* and *in vivo* PD models, and calcitriol was applied in these models. Results showed that parthanatos existed in the MPP^+^-induced cell model and pretreatment with calcitriol improved cell viability, reduced the excessive activation of PARP1, and relieved parthanatos. The application of calcitriol in the MPTP subacute animal model also improved behavioral tests, restored the damage to dopamine neurons, and reduced the activation of PARP1-related signaling pathways. To verify whether calcitriol interacts with PARP1 through its vitamin D receptor (VDR), siRNA, and overexpression plasmids were used to downregulate or overexpress VDR. Following the downregulation of VDR, the expression and activation of PARP1 increased and PARP1 was inhibited when VDR was overexpressed. Coimmunoprecipitation verified the combination of VDR and PARP1. In short, calcitriol can substantially improve parthanatos in the MPP^+^-induced cell model and MPTP model, and the protective effect might be partly through the VDR/PARP1 pathway, which provides a new possibility for the treatment of PD.

## Introduction

Parkinson’s disease (PD) is the second most common neurodegenerative disease, with clinical manifestations of tremors, bradykinesia, rigidity, and postural balance disorders, which seriously affects patient quality of life (Samii et al., 2004). The main pathological hallmarks of PD include degeneration and loss of dopaminergic neurons in the substantia nigra and striatum (Samii et al., 2004). The etiology and mechanism of PD are currently unclear, but recent studies have suggested links between vitamin D and PD (Rimmelzwaan et al., 2016).

Vitamin D functions in the regulation of bone metabolism and calcium homeostasis; unexpected functions of vitamin D in other systems and diseases, such as cardiovascular disease, autoimmune disease, cancer, and neurodegenerative diseases, have recently been found (Zmijewski, 2019). Vitamin D is a fat-soluble steroid that can be obtained through food or exposure to sunlight and is sequentially hydroxylated in the liver and kidneys to become active 1,25-dihydroxyvitamin D3 (calcitriol), which binds to the vitamin D receptor (VDR) to exert its function (Lv et al., 2020). Activated vitamin D can pass through the blood–brain barrier and can be synthesized in the brain (Harms et al., 2011). Immunofluorescence has shown that VDR and vitamin D hydroxylase are expressed most abundantly in the substantia nigra and are related to the development of midbrain dopaminergic neurons, suggesting that vitamin D may be more associated with PD in the central nervous system (Kesby et al., 2011; Orme et al., 2016). A study in Finland found an association between vitamin D levels and a risk of PD (Knekt et al., 2010). In patients with PD, the serum/plasma 25-hydroxyvitamin D levels are lower than in healthy individuals, and lower serum vitamin D levels are related to the severity of motor symptoms in PD (Rimmelzwaan et al., 2016; Sleeman et al., 2017). The use of vitamin D in a PD mouse model can improve behavioral performance and reduce damage to dopaminergic neurons, inflammation (Calvello et al., 2017; Koduah et al., 2017), and active autophagy (Jang et al., 2014). Several partly controversial studies have suggested a connection between vitamins and PD, but the mechanism remains unclear.

Poly (ADP-ribose) (PAR) polymerase-1 (PARP1) is an essential ribozyme that responds to DNA damage and repairs DNA to promote cell survival (Pascal, 2018). However, when DNA is damaged mildly to severely, the overactivation of PARP1 leads to the depletion of nicotinamide adenine dinucleotide (NAD^+^) and ATP in the cells, leading to the excessive synthesis and aggregation of PAR, which leads to apoptosis induction factor (AIF) transfer from the mitochondria to the nucleus and combines with macrophage migration inhibitory factor to cause cell death (Tang et al., 2019). This type of cell death is a distinct process of programmed cell death called PARP1-dependent cell death, also known as parthanatos, which does not depend on caspase and is distinguished from other forms of cell death, such as necrosis and apoptosis (Tang et al., 2019; Park et al., 2020). Previous studies have confirmed the presence of significant PARP1 activation in cell and animal models of PD (Martire et al., 2015; Park et al., 2020). Elevated PAR levels have also been found in the cerebrospinal fluid of patients with PD (Kam et al., 2018).

Vitamin D reduces reactive oxygen species (ROS), which are the activating factors of PARP1 (Berridge, 2016). Calcitriol is a potential PARP1 inhibitor in diabetic nephropathy and cardiomyopathy models as well as macrophage cell lines (Mabley et al., 2007; Qu et al., 2017; Wang et al., 2020), which allows the observation of a possible relationship between vitamin D and PARP1. Calcitriol may also play a protective role by inhibiting PARP1 in the PD model. In this study, we explored parthanatos in 1-methyl-4-phenylpyridinium (MPP^+^)- and 1-methyl-4-phenyl-1,2,3,6-tetrahydropyridine (MPTP)-induced PD models and the effect of calcitriol on parthanatos.

## Materials and Methods

### Plasma Sample Collection

With the approval of Medical Ethics Committee of Tongji Medical College, Huazhong University of Science and Technology, the blood plasma samples used in this study were collected from the Wuhan Union Hospital, Tongji Medical College, Huazhong University of Science and Technology. In this study, 25 patients with PD (mean age 64.8 ± 8.75 years) and 15 age-matched healthy controls (mean age 60.1 ± 4.89 years) were included. All patients were diagnosed with PD by neurologists. Patients with secondary PD, tumors, cerebrovascular diseases, severe heart disease, or chronic inflammation were excluded. The healthy controls were age-matched and had no medical history. All participants were informed of the study, volunteered to participate, and provided written informed consent.

### Enzyme-Linked Immunosorbent Assay

To determine the vitamin D status from the plasma samples of the participants, a 25-hydroxyvitamin D [25(OH) vitamin D] enzyme-linked immunosorbent assay (ELISA) kit (Abcam) was used according to the manufacturer’s instructions.

### Cell Culture

SH-SY5Y cells were cultured in Dulbecco’s modified Eagle’s medium/F12 (Gibco) with 10% fetal bovine serum (Gibco) and 1% penicillin/streptomycin in 5% CO_2_ at 37°C, as previously described (Hu et al., 2019). MPP^+^ (Sigma) was added to the SH-SYSY cells to induce the PD cell model. SH-SY5Y cells were pretreated with calcitriol (MedChemExpress) or control for 4 h then exposed to MPP^+^ for 24 h.

### Cell Counting Kit-8 Assay

The Cell Counting Kit-8 (CCK8) assay (Dojindo Technology) was conducted as previously described (Hu et al., 2019). Briefly, cells were seeded into a 96-well plate and treated as described in cell culture. Each well was then incubated with 10% CCK-8 solution for 1.5 h, and the absorbance of each well was read at 450 nm using a microplate reader.

### Protein Extract and Western Blotting

Total proteins from cells were extracted using RIPA lysis buffer according to the manufacturer’s instructions. Nuclear and cytosolic fractions were extracted from SH-SY5Y cells using a cytoplasmic and nuclear protein extraction kit (Beyotime Biotechnology). A bicinchoninic acid protein assay kit (Beyotime Biotechnology) was used to determine the protein concentration. Western blotting was performed as described previously (Hu et al., 2019). Briefly, 20–40 μg of protein was added to a 10–12% sodium dodecyl sulfate-polyacrylamide gel electrophoresis gel. After electrophoresis, the proteins on the gel were transferred onto nitrocellulose membranes (Millipore). The primary antibody was incubated overnight after blocking with 5% non-fat milk for 1 h. The primary antibodies were diluted as follows: VDR antibody (1:1,000, Cell Signaling Technology), PARP1 antibody (1:2,000, Proteintech), PAR antibody (1:1,000, Trevigen), AIF antibody (1:1,000, Proteintech), cleaved-PARP1 antibody (1:1,000, Abcam), tyrosine hydroxylase (TH) antibody (1:2,000, Proteintech), phospho-histone H2A.X (Ser139) antibody (1:1,000, Cell Signaling Technology), β-actin antibody (1:5,000, Proteintech), glyceraldehyde-3-phosphate dehydrogenase (GAPDH) antibody (1:5,000, Proteintech), and histone H3 antibody (1:1,000, Proteintech). Next, part of the membrane was incubated with horseradish peroxidase (HRP)-conjugated rabbit or mouse secondary antibody for 1 h, washed three times, and soaked with enhanced chemiluminescence solution, and the Bio-Rad imaging system was used for exposure. Other membranes were incubated with a dylight800 goat anti-rabbit or mouse secondary antibody and recorded using the Odyssey infrared laser imaging system. Outcomes were analyzed using ImageJ software.

### Small Interfering RNA and Plasmid Transfection

Cells were cultured in six-well plates until they reached 50% confluence. Fifty nanomoles of VDR or control small interfering RNA (siRNA, RiboBio) with transfection reagent Lipo2000 (Invitrogen) were then added to the SH-SY5Y cells according to the manufacturer’s instructions. A 2-μg human VDR overexpression plasmid or control plasmid (Genechem) with transfection reagent neofect (Neofect) was also added to the SH-SY5Y cells. Next, 48–72 h after transfection, cells were collected for real-time PCR or western blotting.

### Real-Time PCR

RNA extraction and reverse transcription were performed according to the manufacturer’s instructions. SYBR Green Master Mix (Takara), primers, and cDNA were added to the PCR plates, and the reaction was carried out in a real-time PCR system (StepOnePlus real-time PCR System). The following primers were used: VDR 5-GTGGACATCGGCATGATGAAG-3 (forward) and 5-GGTCGTAGGTCTTATGGTGGG-3 (reverse); PARP1 5-CGGAGTCTTCGGATAAGCTCT-3 (forward), and 5-TTTCCATCAAACATGGGCGAC-3 (reverse); β-actin 5-AGA GCTACGAGCTGCCTGAC-3 (forward) and 5-AGCACTGT GTTGGCGTACAG-3 (reverse).

### Measurement of ROS

Briefly, the 2,7-dichlorodi-hydrofluorescein diacetate (DCFH-DA) fluorescent probe (Beyotime Biotechnology) was loaded to the cells after treatment, and the intracellular levels of ROS were measured by flow cytometry. The ROS production proportion and mean fluorescence intensity were then analyzed using FlowJo-V10.

### Measurement of NAD^+^ Level

Cells were treated as previously described above in cell culture part. NAD^+^ extraction buffer (Beyotime Biotechnology) was added to each plate, centrifuged, and heated according to the manufacturer’s instructions. After 2-(2-methoxy-4-nitrophenyl)-3-(4-nitrophenyl)-5-(2,4-disulfophenyl)-2H-tetrazolium monosodium salt (WST-8) incubation, the absorbance was measured at 450 nm using a microplate reader.

### Animals and Treatment

All mouse experiments complied with the NIH guidelines for the Care and Use of Laboratory Animals and followed the ethical code of the Laboratory Animal Ethics Committee of Huazhong University of Science and Technology to minimize animal discomfort during the experiment. The 8-week-old male C57/BL6 mice were obtained from the Beijing Vital River and raised in the specific pathogen-free animal room of the Experimental Animal Center of Huazhong University of Science and Technology. The ambient temperature was 21–23°C, and the light cycle was 12 h dark/light. All mice had free access to food and water. C57/BL6 mice were randomly divided into four groups (*n* = 10 per group) as follows: MPTP group, injected with MPTP (Sigma, 30 mg/kg/day, i.p.) for 7 days; calcitriol + MPTP group, injected with calcitriol (2.5 μg/kg/day, i.p.) commencing 7 days prior to MPTP administration and lasting for 21 days; calcitriol group, administered calcitriol (2.5 μg/kg/day, i.p.) for 21 days; and control group, administered solvent control.

### Behavioral Assays

#### Rotarod Testing

Rotarod testing was conducted as previously described (Guo et al., 2019). The mice were placed on the rotarod, accelerated to 30 rpm, and rotated for 5 min. Latency to fall time was recorded. Practice runs took place for 3 days prior to the actual experiment to allow the mice to adapt.

#### Pole Testing

A vertical pole of 50 cM in length and 1 cM in diameter was used. The outer surface was wrapped with gauze to prevent slipping. Each mouse was placed at the top of the pole, and the time taken for the mouse to travel from the top to the bottom was recorded. If the mouse remained on the pole for more than 1 min or fell halfway, this was recorded as 60 s.

### Immunofluorescent Staining

Immunofluorescent staining was performed on paraffin-embedded sections. After deparaffinization and hydration, antigen retrieval was conducted using a citric acid solution (pH 6) or ethylene diamine tetraacetic acid solution (pH 9) (according to primary antibody instructions) and heating for 8 min. A 3% hydrogen peroxide solution was used to inactivate endogenous peroxidase, and sections was then blocked with 5% bovine serum albumin and incubated with the primary antibody at 4°C overnight. The primary antibodies were diluted as follows: VDR antibody (1:100, Cell Signaling Technology), PARP1 antibody (1:200, Proteintech), PAR antibody (1:100, Trevigen), AIF antibody (1:100, Proteintech), and TH antibody (1:500, Abcam). A secondary antibody conjugated to Alexa Fluor 488 or 594 was then added and nuclei were stained with 4,6-diamidino-2-phenylindole (DAPI). The sections were observed under a fluorescence microscope. Cells were seeded onto coverslips and treated as previously described above in cell culture. After fixing and permeabilization, the staining procedure was undertaken as described above from blocking.

### TdT-Mediated dUTP Nick-End Labeling Staining

Paraffin-embedded sections were deparaffinized and hydrated then treated with 20 μg/ml proteinase K. A TdT-mediated dUTP nick-end labeling (TUNEL) assay kit (Roche) was used in these sections, and the nuclei were stained with DAPI. For cell TUNEL staining, coverslips were fixed and permeabilized, and the TUNEL assay kit was used and the nuclei were stained.

### Immunohistochemistry Staining

The immunohistochemical staining procedure for paraffin sections was the same as that for immunofluorescence staining until primary antibody incubation. The primary antibodies were diluted as follows: cleaved-PARP1 antibody (1:100, Abcam) and TH antibody (1:500, Proteintech). The HRP-labeled secondary antibody was subsequently incubated, followed by the diaminobenzidine reaction. Nuclei were stained with hematoxylin as needed. After transparency, the images from the sections were examined under a microscope.

### Statistical Analysis

All values were displayed as mean + SEM using the GraphPad Prism 8 software. Data were analyzed using an unpaired two-tailed *t* test or one-way analysis of variance. *P* < 0.05 was considered to indicate significance.

## Results

### Plasma 25(OH) Vitamin D Decrease and PARP1 Activation in Patients With PD

**FIGURE 1:**
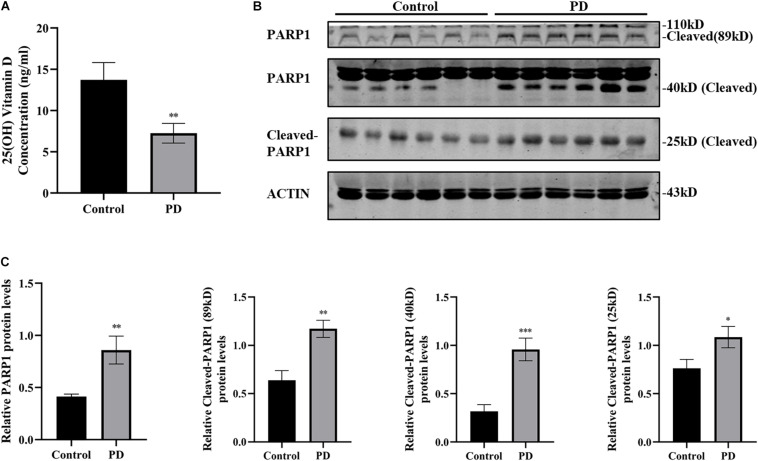
Plasma 25(OH) vitamin D decrease and PARP1 activation in patients with PD. 25(OH) vitamin D concentrations in plasma determined by ELISA **(A)**. (PD patients, *n* = 25; healthy controls, *n* = 15). The expressions of PARP1 and cleaved-PARP1 fragments of 89, 40, and 25 kD in plasma were detected by western blotting **(B)**. Quantification of PARP-1 expressions and cleaved-PARP1 expression **(C)**. (*n* = 6). Data are shown as mean ± SEM, *p < 0.05, **p < 0.01, ***p < 0.001.

The most common way to determine the body’s vitamin D status is to measure the concentration of 25(OH) vitamin D in serum or plasma. A 25(OH) vitamin D ELISA kit was used to measure plasma 25(OH) vitamin D levels in patients with PD and healthy controls. Results showed that the plasma 25(OH) vitamin D levels of patients with PD (7.24 ± 1.192 ng/ml) were substantially lower than those of the healthy controls (13.71 ± 2.101 ng/ml) (Figure 1A). Next, the plasma PARP1 levels and cleaved PARP1 of the patients with PD were detected by western blotting, and the results indicated that 89, 40, and 25 kD of cleaved PARP1 in the plasma of patients with PD increased, which reflects the activation of PARP1 (Figures 1B,C).

### Activated Parthanatos and Reduced VDR Expression in MPP^+^-Induced SH-SY5Y Cells

**FIGURE 2:**
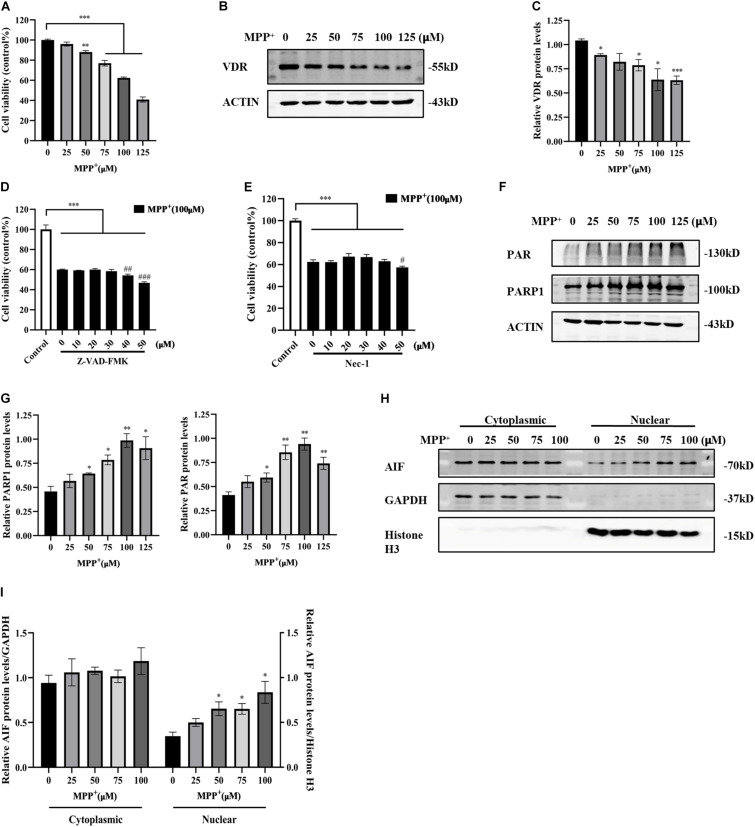
Reduced VDR expression and activated parthanatos in MPP^+^-induced SH-SY5Y cells. SH-SY5Y cells were treated with different concentrations of MPP^+^ for 24 h. Cell viability was assessed by CCK8 assay **(A)**. VDR expression was detected by western blotting **(B)** and quantified **(C)**. SH-SY5Y cells were pretreated with different concentrations of Z-VAD-FMK for 1 h before 100 μM MPP^+^ treatment, then CCK8 assay assessed cell viability **(D)**. Compared with control, ***p < 0.001; compared with MPP^+^ group, ^##^*p* < 0.01, ^###^*p* < 0.001, *n* = 5. SH-SY5Y cells were pretreated with different concentrations of Nec-1 for 4 h before 100 μM MPP^+^ treatment, then CCK8 assay assessed cell viability **(E)**. Compared with control, ***p < 0.001; compared with MPP^+^ group, ^#^*p* < 0.05, *n* = 5. SH-SY5Y cells were treated with different concentrations of MPP^+^ for 24 h. PAR and PARP1 expression were detected by western blotting **(F)** and quantified **(G)**. The cytoplasmic and nuclear proteins were extracted separately. AIF expression was detected by western blotting **(H)** and quantified **(I)**. Data are shown as mean ± SEM, based on three independent experiments, *p < 0.05, **p < 0.01, ***p < 0.001.

MPP^+^ is a classical neurotoxin widely used in PD cell models that causes mitochondrial dysfunction and ROS production, thereby causing neuronal death. Parthanatos is a form of programmed cell death that can be triggered by the accumulation of ROS or DNA damage. It is not currently clear whether MPP^+^ can engender parthanatos. Here, SH-SY5Y cells were exposed to different concentrations of MPP^+^ to determine the optimal concentration for inducing cell death. Cell viability gradually decreased with increasing MPP^+^ concentration (Figure 2A). The apoptosis inhibitor Z-VAD-FMK and necroptosis inhibitor necrostatin-1 (Nec-1) did not reverse the decrease in cell viability caused by MPP^+^ (Figures 2D,E), suggesting that MPP^+^-induced cell death did not depend on caspase or necroptosis. As the concentration of MPP^+^ treatment increased, the levels of PAR and PARP1 also increased (Figures 2F,G). The accumulation of PAR triggered the translocation of AIF from the mitochondria to the nucleus, which eventually caused cell death. Cytoplasmic and nuclear proteins were extracted separately. GAPDH was used as the internal control of cytoplasmic protein. Histone H3 was used as the internal control of nuclear protein. The nuclear translocation of AIF was enhanced with increasing MPP^+^ concentration (Figures 2H,I). Overall, 100 μM MPP^+^ treatment was most obvious in parthanatos, and this concentration was selected for the subsequent experiments. At the same time, the expression of VDR decreased significantly as the concentration of MPP^+^ increased (Figures 2B,C).

### Calcitriol Protected SH-SY5Y Cells From Parthanatos

**FIGURE 3:**
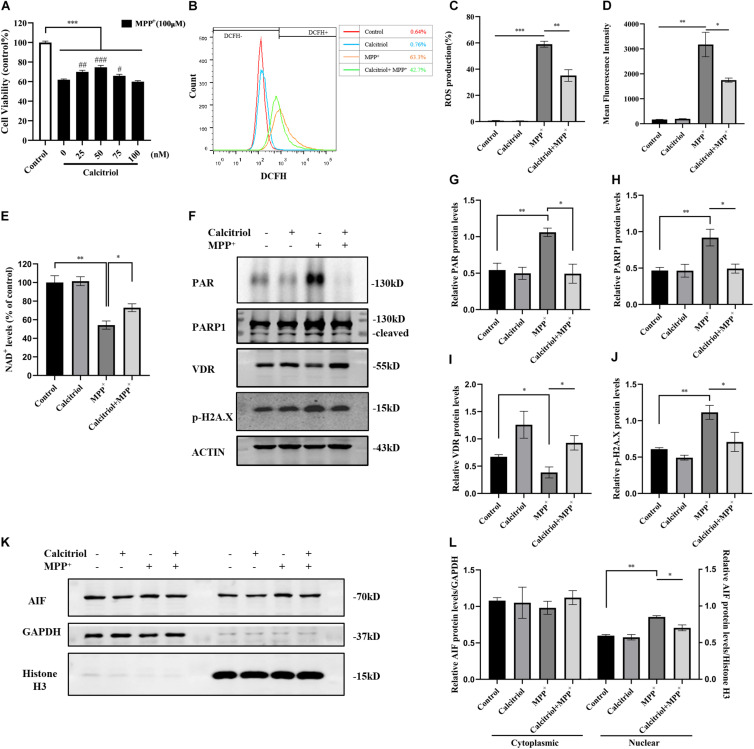
Calcitriol protected SH-SY5Y cells from parthanatos. SH-SY5Y cells were pretreated with different concentrations of calcitriol for 4 h before 100 μM MPP^+^ treatment, then CCK8 assay assessed cell viability. Compared with control, ***p < 0.001; compared with MPP^+^ group, ^#^*p* < 0.05, ^##^*p* < 0.01, ^###^*p* < 0.001, *n* = 10 **(A)**. ROS production assessed by FACS analysis **(B)**. Quantification of ROS production **(C)** and mean fluorescence intensity **(D)** in all groups, *n* = 3. Compared with control, *p < 0.05, **p < 0.01, ***p < 0.001. NAD^+^ levels were assessed by WST-8 in all groups, *n* = 3. Compared with control, *p < 0.05, **p < 0.01 **(E)**. PAR, PARP1, VDR, and phosphor-H2A.X expression were detected by western blotting **(F)** and quantified **(G–J)**. Analysis translocation of AIF by western blotting **(K)** and quantified **(L)**. Data are shown as mean ± SEM, based on three independent experiments, *p < 0.05, **p < 0.01, ***p < 0.001.

**FIGURE 4:**
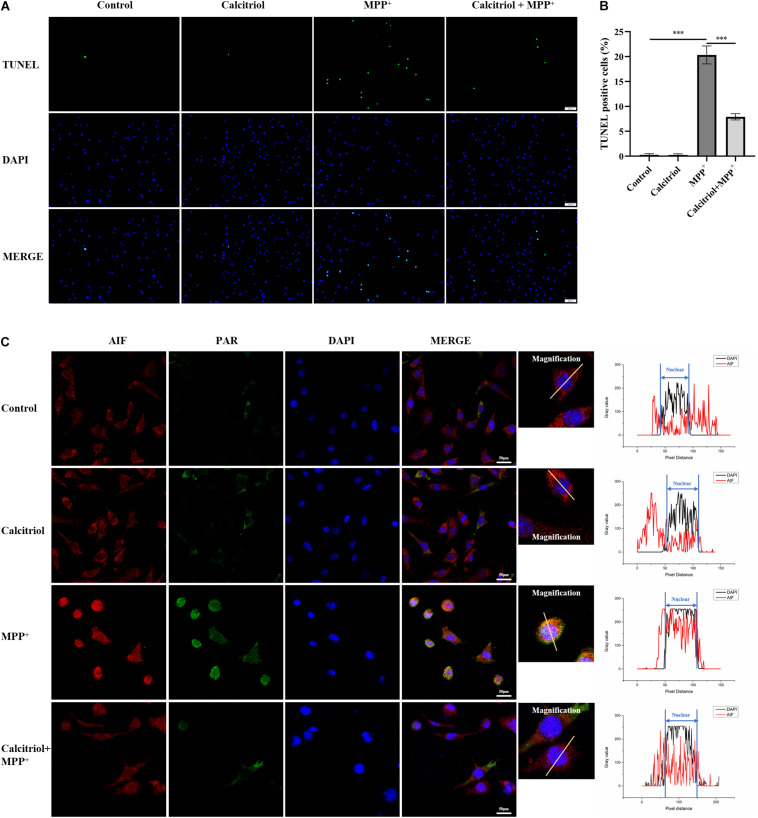
Calcitriol alleviated DNA damage, PAR accumulation, and AIF nuclear translocation in MPP^+^-induced cells. TUNEL stain detected by fluorescence microscopy [**(A)**, scale bar, 100 μm]. For recording the proportion of TUNEL-positive cells, five field of views were randomly selected in each group and the average value was calculated. Data are shown as mean ± SEM, based on three independent experiments, ***p < 0.001 **(B)**. Representative images of PAR and AIF by fluorescence microscopy. Cell coverslips were costained with PAR (green), AIF (red), and DAPI (blue) [**(C)**, scale bar, 50 μm]. Observed AIF translocation by analyzing the fluorescence intensity of the two channels (AIF and DAPI).

Some vitamin D analogs have neurotrophic and inflammation-reducing effects and can upregulate the expression of its receptor VDR. Among them, the active form of vitamin D, calcitriol, was selected for this study. The results demonstrated that 25–75 nM calcitriol ameliorated the cell viability decline caused by MPP^+^ treatment (Figure 3A), the concentration of 50 nM show the most obvious effect, and this concentration was selected for subsequent experiments. We determined ROS levels in MPP^+^-treated SH-SY5Y cells using the DCFH-DA probe. As shown in Figures 3B–D, MPP^+^ treatment significantly increased ROS production and calcitriol pretreatment alleviated ROS production. MPP^+^ treatment led to a decline in NAD^+^ levels, reflecting the excessive consumption of NAD^+^ (Figure 3E). The results showed that pretreatment with calcitriol significantly increased the expression of VDR. Meanwhile, calcitriol pretreatment reduced the accumulation of PAR and the activation of PARP1 and phosphor-histone H2A.X (Ser139), as well as the nuclear translocation of AIF in MPP^+^-induced cells (Figures 3F–L). In addition to detecting the expression of phosphor-H2A.X by western blotting, TUNEL staining was performed to observe DNA damage, which is the activation factor of PARP1. As shown in Figures 4A,B, TUNEL-positive cells increased significantly after MPP^+^ treatment and calcitriol pretreatment alleviated this effect. To confirm the western blotting results, we performed immunofluorescence staining on the cells, which showed similar results in accumulation of PAR and nuclear translocation of AIF (Figure 4C). We used ImageJ software for fluorescence colocalization analysis. After MPP^+^ treatment, AIF expression increased in the nucleus and calcitriol pretreatment reduced the nuclear translocation of AIF.

### The Interaction Between VDR and PARP1

**FIGURE 5:**
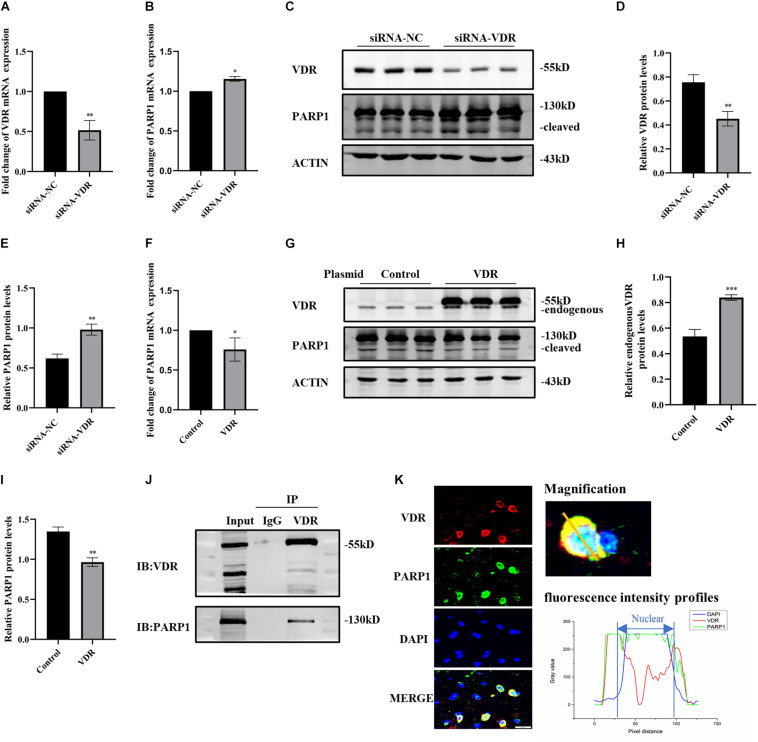
The interaction between VDR and PARP1. SH-SY5Y cells were transferred with siRNA of VDR or negative control; VDR and PARP1 levels were assessed by real-time PCR **(A,B)** and western blotting **(C)**. Quantification of VDR **(D)** and PARP-1 **(E)** expressions. SH-SY5Y cells were transferred with VDR overexpression plasmid or negative-control plasmid; PARP1 levels were assessed by real-time PCR **(F)**. VDR and PARP1 levels were assessed by western blotting **(G)** and quantified **(H,I)**. Coimmunoprecipitation shows the binding of VDR and PARP1 **(J)**. Fluorescence colocalization analysis of VDR and PARP1 in sections **(K)**. Data are shown as mean ± SEM, based on three independent experiments, *p < 0.05, **p < 0.01, ***p < 0.001.

Since calcitriol combined with its receptor VDR exerts its function, we investigated the inhibitory effect of calcitriol on PARP1 through VDR. First, SH-SY5Y cells were transfected with VDR siRNA or negative control, and real-time PCR and western blotting were used to verify the knockdown effect (Figures 5A,C,D). The RNA and protein levels of PARP1 increased significantly after VDR siRNA transfection (Figures 5B,C,E). The VDR overexpression plasmid or control plasmid was then used to transfect SH-SY5Y cells. Western blotting revealed the upregulation effects of VDR (Figures 5G,H). The RNA and protein levels of PARP1 significantly decreased after VDR plasmid transfection (Figures 5F,G,I). The results showed that the upregulation or downregulation of VDR had a significant impact on PARP1 expression. Next, coimmunoprecipitation was performed to determine whether VDR and PARP1 were directly combined. Figure 5J shows that VDR and PARP1 did combine. VDR and PARP1 also showed fluorescence colocalization in tissue sections (Figure 5K).

### Calcitriol Alleviated Behavioral and Dopaminergic Neuron Damage in a MPTP Model of PD

**FIGURE 6:**
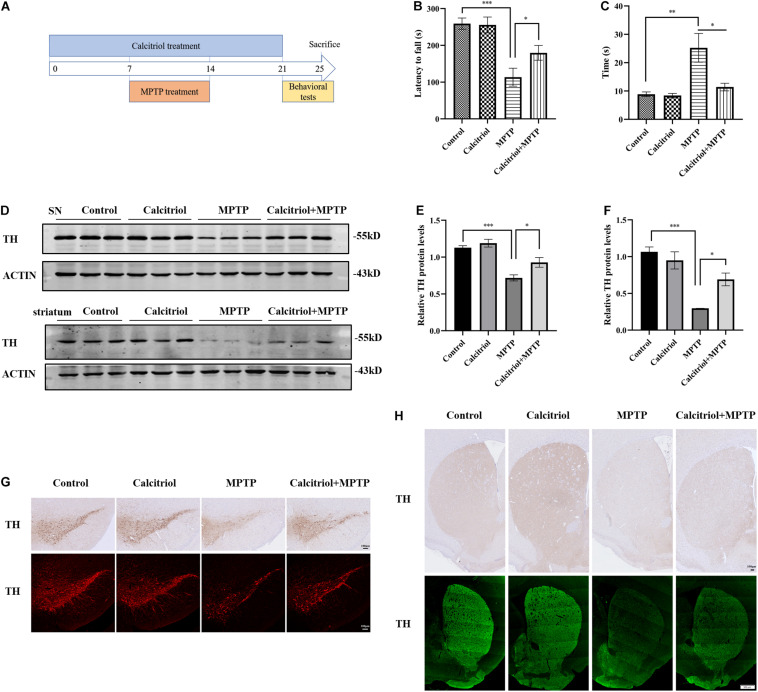
Calcitriol alleviated behavioral and dopaminergic neuron damage in a MPTP model of PD. Schematic diagram of the MPTP model experimental design **(A)**. Performance on the rotarod **(B)** and pole test **(C)** (*n* = 10). TH expression was detected by western blotting in substantia nigra (SN) and striatum **(D)**. TH expression was quantified in SN **(E)** and striatum **(F)**. Representative images of TH immunofluorescence and immunochemistry of substantia nigra pars compacta [**(G)**, scale bar, 100 μm]. Representative images of TH immunofluorescence and immunochemistry of striatum [**(H)**, upper image scale bar, 100 μm; lower image scale bar, 500 μm]. Data are shown as mean ± SEM, *p < 0.05, **p < 0.01, ***p < 0.001.

The role of calcitriol was investigated in the MPP^+^ cell model before focusing on whether calcitriol had protective effects in the PD animal model. For this, the MPTP subacute model was selected. C57/BL6 mice were randomly divided into four groups as follows: control, calcitriol, MPTP, and calcitriol + MPTP. A schematic diagram of the MPTP model experimental design is shown in Figure 6A. After the final administration, behavioral rotarod and pole tests were carried out. As shown in Figure 6B, the MPTP group had a decrease in latency time to falling from the rod compared with the control group in the rotarod test, and calcitriol treatment reversed this effect. In the pole test, the total time taken to climb down the pole in the MPTP group was significantly extended compared with that of the control group, while calcitriol treatment shortened this (Figure 6C). TH is a dopaminergic neuronal-specific marker. Western blotting detected the TH protein expression of substantia nigra. The level of TH protein in the MPTP group was significantly decreased compared with that in the control group and calcitriol + MPTP group (Figures 6D,E). Striatum protein showed similar results (Figures 6D,F). The immunofluorescence and immunohistochemical staining of TH demonstrated consistent results with western blotting results in the substantia nigra (Figure 6G) and striatum (Figure 6H).

### Calcitriol Alleviated Parthanatos in the MPTP Model

**FIGURE 7:**
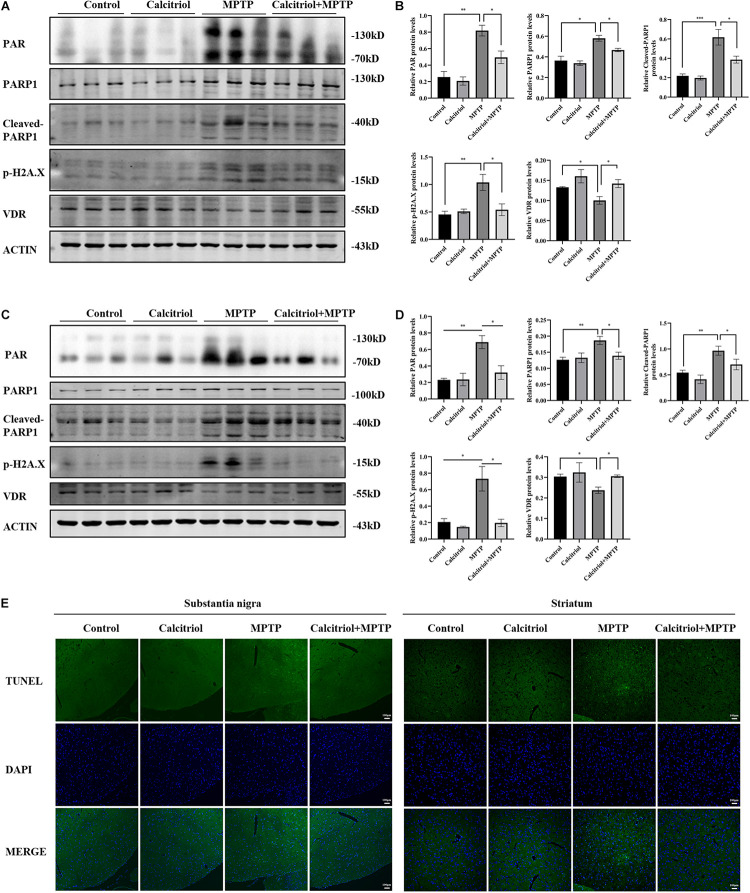
Calcitriol alleviated PARP1 signaling pathway *in vivo*. PAR, PARP1, cleaved-PARP1, phosphor-H2A.X, and VDR levels in substantia nigra were assessed by western blotting **(A)** and quantified **(B)**. PAR, PARP1, cleaved-PARP1, phosphor-H2A.X, and VDR levels in striatum were assessed by western blotting **(C)** and quantified **(D)**. Representative immunofluorescence images of TUNEL staining of substantia nigra and striatum [**(E)**, scale bar, 100 μm]. Data are shown as mean ± SEM, based on three independent experiments, *p < 0.05, **p < 0.01, ***p < 0.001.

**FIGURE 8:**
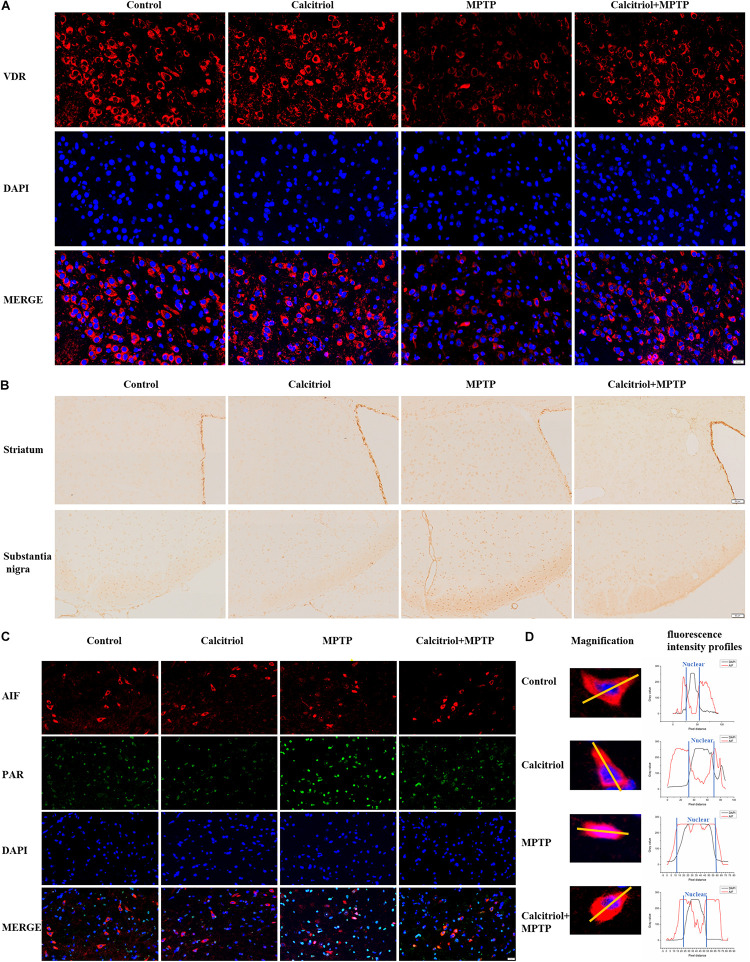
Calcitriol increased VDR and alleviated parthanatos in sections of MPTP models. Representative images of VDR immunofluorescence of substantia nigra [**(A)**, scale bar, 50 μm]. Representative images of cleaved PARP1 immunochemistry of striatum and substantia nigra [**(B)**, scale bar, 50 μm]. Representative images of PAR and AIF immunofluorescence of substantia nigra [**(C)**, scale bar, 20 μm]; then the selected representative magnification images for fluorescence intensity profile analysis of the two channels (AIF and DAPI) **(D)**. The experiments were repeated three times.

Next, we focused on whether calcitriol affected parthanatos in the MPTP model. Western blotting showed that the MPTP group had higher PAR, PARP1, cleaved-PARP1, and phosphor-H2A.X levels than the control group in the substantia nigra (Figures 7A,B) and striatum (Figures 7C,D), calcitriol mitigated the levels of these proteins in the parthanatos signaling pathway. In the substantia nigra (Figures 7A,B) and striatum (Figures 7C,D), VDR protein levels decreased in the MPTP group, and calcitriol treatment recovered them. TUNEL staining was performed to observe DNA damage, and the positive staining of TUNEL in the MPTP group increased significantly in the brain sections of the substantia nigra or striatum, and calcitriol treatment reduced this (Figure 7E), showing a protective effect against DNA damage. To further confirm western blotting analysis, VDR was detected by immunofluorescence confocal microscopy in the substantia nigra sections, which was consistent with the western blotting results (Figure 8A). As shown in Figure 8B, immunohistochemical staining of cleaved PARP1 in the substantia nigra and striatum sections also showed an increase in cleaved PARP1 in the MPTP group, and calcitriol treatment reduced it, reflecting the inhibitory effect of calcitriol on PARP1 activation. Finally, we conducted PAR and AIF immunofluorescence staining in the substantia nigra, and the results showed that the PAR increased in the MPTP group and that calcitriol treatment reversed this effect (Figure 8C). In addition, the localization of AIF in the nucleus significantly increased in the Figure 8C. We performed fluorescence intensity profile analysis to quantify the increase of AIF in the nucleus (Figure 8D).

## Discussion

In recent years, the relationship between PD and vitamin D has gradually attracted attention. Patients with PD have lower serum/plasma 25(OH) vitamin D concentrations than healthy controls (Evatt et al., 2011; van den Bos et al., 2013; Petersen et al., 2014; Rimmelzwaan et al., 2016). Vitamin D levels are negatively correlated with PD severity (Suzuki et al., 2012; Luo et al., 2018), and vitamin D may be a prognostic biomarker of PD (Lawton et al., 2020). There have been limited and controversial studies regarding whether vitamin D is a risk factor for PD. Some believe that the decrease in vitamin D levels may be the result of PD due to dyskinesia and gastrointestinal symptoms, while others believe that vitamin D deficiency may be related to the etiology of PD (Newmark and Newmark, 2007; Wang et al., 2015; Fullard et al., 2017). Next, we focused on clinical trials of vitamin D supplementation in patients with PD. A clinical trial revealed that vitamin D supplementation significantly improved the H&Y stage and UPDRS score compared with placebo in patients with PD (Suzuki et al., 2013). These studies show a possible association between vitamin D and PD. Studies regarding the protective effects and mechanisms of vitamin D in PD models are very limited at present. We selected calcitriol, the final active form of vitamin D, to examine the mechanism of vitamin D in PD models *in vitro* and *in vivo*. We first verified the plasma 25(OH) vitamin D levels in patients with PD, and the results showed that the 25(OH) vitamin D level of these patients was significantly lower than that of healthy controls, which is consistent with the results of previous studies. We verified that the expression level of VDR was reduced in MPP^+^-treated SH-SY5Y cells.

1-methyl-4-phenyl-1,2,3,6-tetrahydropyridine and its metabolite MPP^+^ are neurotoxic, can inhibit mitochondrial complex I, block the respiratory chain, interrupt cell energy supply, and cause ROS production and cell death (Nicklas et al., 1987). DNA damage, ROS, and pathological proteins are all strong activating factors of PARP1 (Park et al., 2020). Cell death caused by the activation of PARP1 and PAR polymer accumulation is called parthanatos, which is different from the traditional form of cell death, and apoptosis inhibitors, such as Z-VAD-FMK, cannot inhibit it (Park et al., 2020). The results of previous studies support the activation of PARP1 and accumulation of PAR in the α-syn pathologic PD model (Kam et al., 2018). PAR in the cerebrospinal fluid of patients with PD is significantly higher than that in controls (Kam et al., 2018). MPTP models also cause PARP1 activation, and the application of genetic knockout or PARP1 inhibitors can reduce PD pathology (Yokoyama et al., 2010). We found that PARP1 in the plasma of patients with PD patients was significantly activated. In addition to the common cleavage-PARP1 fragments 25 and 89 kD, cleaved-PARP1 fragments around 40 kD in human plasma are obvious. However, the role of the 40-kD cleavage fragment is not clear. Studies have shown that this fragment is mainly cleaved by calpain, which is initiated by intracellular calcium levels (Chaitanya et al., 2010). The increase of intracellular calcium can activate calpain and increase ROS. Both ROS activation and calpain activation can activate PARP1, and calpain can cleave PARP1 to produce 40 kD fragments (Chaitanya et al., 2010). All the cleavage fragment of PARP1 reflects the activation of PARP1 and is the initial step of parthantos. First, we verified whether parthanatos existed in the MPP^+^-induced SH-SY5Y cell model. The results showed that MPP^+^ caused PARP1 activation, PAR aggregation, and AIF translocation to the nucleus in a concentration-dependent manner. Z-VAD-FMK and nec-1 usage could not reverse the cytotoxicity caused by MPP^+^, which was consistent with the characteristics of parthanatos. This result was supported by those of a study by Nho et al. (2020).

The mechanism of calcitriol is mainly focused on its neuroprotective and anti-inflammatory effects in the PD model. Calcitriol can increase the expression of glial-cell-line-derived neurotrophic factor (GDNF) and other dopaminergic neuron-related nutritional factors, and directly increases the expression of TH (Cui et al., 2015; Pertile et al., 2018). In terms of anti-inflammatory effects, it mainly shows reducing effects on tumor necrosis factor alpha, microglial activation, and ROS production (Jang et al., 2015; Calvello et al., 2017). Some studies also suggested the role of calcitriol in autophagy (Lyu et al., 2020), calcitriol reduces rotenone and MPTP-mediated neurotoxicity by enhancing autophagy-related signaling pathways (Jang et al., 2014; Li et al., 2015). PARP1 inhibitors have been shown to reduce the spread of synuclein and reduce the pathology of PD. Calcitriol is a potential PARP1 inhibitor in macrophage cell lines, and the association of vitamin D and PARP1 occurs in diabetic cardiomyopathy and nephropathy models (Mabley et al., 2007; Qu et al., 2017; Wang et al., 2020). In a diabetes model simulated by incubating with fetal bovine serum in Schwann cells, calcitriol treatment can reduce cell apoptosis and reduce cleavage of PARP1 (Xu et al., 2019). Vitamin D has not yet been linked to PARP1 or parthanatos in the PD model.

In terms of PARP1 activation, calcitriol can inhibit the stimuli of PARP1 activation by inhibiting intracellular calcium overload, ROS, and reduce cell death. Its effect of reducing cell death can be achieved by activating autophagy, neurotrophic, and anti-inflammatory effects (Jang et al., 2014, 2015; Pertile et al., 2018; Lyu et al., 2020). In this study, we investigated the effect of calcitriol on parthanatos. Our results show that pretreatment with calcitriol significantly reduces ROS production, PARP1 activation, PAR accumulation, and DNA damage caused by MPP^+^ in SH-SY5Y cells, and partly restores the excessive consumption of NAD^+^. Our results support the relieving effect of calcitriol on parthanatos. Calcitriol increases the expression of GDNF and nuclear factor erythroid-2-related factor 2 to protect cells from damage caused by ROS, which is in accordance with our results (Pertile et al., 2018; Lin et al., 2020). Next, we verified the role of calcitriol in the MPTP mouse model. Calcitriol treatment significantly improved the behavior of MPTP animal models and partly restored TH. Similar to *in vitro* experiments, calcitriol also reduced DNA damage and PARP1-related parthanatos signaling pathways in MPTP models.

Calcitriol regulates other genes through its receptors, VDR, and upregulates the expression of VDR. 6-Hydroxydopamine or preformed fibrils cause transcriptional inhibition of VDR and its target genes (P-glycoprotein), calcitriol significantly restore the expression of VDR and P-glycoprotein, reflecting the role of vitamin D in maintaining vascular balance in PD (Kim et al., 2020). This also confirms the role of calcitriol in other animal models of PD. The reduction of VDR expression caused by MPP^+^ and MPTP was also observed in our experiments, and calcitriol treatment recovered it. With the upregulation of the VDR gene, PARP1 level is downregulated, and with the downregulation of the VDR gene, PARP1 is upregulated and activated. Coimmunoprecipitation and fluorescence colocalization analyses showed the combination of VDR and PARP1. We will conduct further studies in the future to confirm our results.

In conclusion, we found that MPP^+^ and MPTP can cause parthanatos and calcitriol can alleviate this through the VDR/PARP1 pathway, showing a new mechanism of calcitriol and providing new insights into the pathogenesis and treatment of PD.

## Data Availability Statement

The original contributions presented in the study are included in the article, further inquiries can be directed to the corresponding author.

## Ethics Statement

The studies involving human participants were reviewed and approved by Medical Ethics Committee of Tongji Medical College, Huazhong University of Science and Technology. The patients/participants provided their written informed consent to participate in this study. The animal study was reviewed and approved by the Laboratory Animal Ethics Committee of Huazhong University of Science and Technology.

## Author Contributions

JH contributed to the conception of the study, conducted experiments, and wrote the manuscript. JW performed data analysis. LK, SY, YS, YL, and QZ helped partly in the experiments. TW was responsible for revising the manuscript, presenting constructive discussions. All authors read and approved the final manuscript.

## Conflict of Interest

The authors declare that the research was conducted in the absence of any commercial or financial relationships that could be construed as a potential conflict of interest.

## Publisher’s Note

All claims expressed in this article are solely those of the authors and do not necessarily represent those of their affiliated organizations, or those of the publisher, the editors and the reviewers. Any product that may be evaluated in this article, or claim that may be made by its manufacturer, is not guaranteed or endorsed by the publisher.
